# Epidemiological intelligence community network intervention: a community response for COVID-19 community transmission

**DOI:** 10.1186/s12889-023-15727-3

**Published:** 2023-06-01

**Authors:** Melissa Marzan-Rodríguez, Kamalich Muniz-Rodriguez, Luisa M. Morales, Iris S. Martínez, Natasha Torres-Borrero, Eida M. Castro-Figueroa

**Affiliations:** 1 Public Health Program, Ponce Health Sciences University, Ponce, PR Puerto Rico; 2 Ponce Research Institute, Ponce Health Sciences University, Ponce, PR Puerto Rico; 3 School of Behavioral Sciences, Ponce Health Sciences University, Ponce, PR Puerto Rico

**Keywords:** Community engagement, COVID-19 response, Capacity building, Community leaders

## Abstract

**Background:**

Expanding and providing access to early detection of severe acute respiratory syndrome coronavirus 2 (SARS-CoV-2) through testing community-based strategies among socially vulnerable communities (SVC) are critical to reducing health disparities. The Epidemiological Intelligence Community Network (EpI-Net) community-based intervention sought to increase coronavirus 2019 (COVID-19) testing uptake and prevention practices among SVC in Puerto Rico (PR). We evaluated EpI-Net’s community leaders’ capacity-building component by assessing pre-post COVID-19 public health workshops’ tests’ score changes and satisfaction among trained community leaders.

**Methods:**

A total of 24 community leaders from SVC in PR have completed four community workshops. Pre- and post-assessments were completed as part of the health promotors training program to evaluate participants’ tests score changes and satisfaction outcomes.

**Results:**

Preliminary results showed: (1) high intervention retention levels of community leaders (85.7% acceptance rate); (2) change in post-test scores for community engagement strategies (p = 0.012); (3) change in post-test educational scores in COVID-19 prevention practices (p = 0.014); and (4) a change in scores in public health emergency management strategies (p < 0.001).

**Conclusions:**

The overall workshop satisfaction was 99.6%. Community leaders have shown the importance of community capacity building as a key component for intervention feasibility and impact.

**Trial registration:**

Our study was retrospectively registered under the ClinicalTrial.gov ID NCT04910542.

**Supplementary Information:**

The online version contains supplementary material available at 10.1186/s12889-023-15727-3.

## Background

The coronavirus 2019 (COVID-19) pandemic has disproportionally affected socially vulnerable communities (SVC) [1–5]. For example, in communities with greater social vulnerability rates, a higher COVID-19 case fatality rate was observed [5]. According to the Centers for Disease Control and Prevention’s Social Vulnerability Index (CDC SVI), socially vulnerable communities are those exposed to stressors caused by natural or human-caused disasters (e.g., earthquakes, hurricanes, pollution) or disease outbreaks (e.g., COVID-19 pandemic) that impact the health of a given community [6, 7]. The CDC SVI is a key tool for planning services among communities with a high potential of experiencing health disparities during a public health emergency (PHE).

In Puerto Rico, SVCs are low socioeconomic status sectors with high rates of psychosocial distress due to natural disaster exposure, air pollution generated by Saharan Dust Storms, and disease outbreaks, such as the COVID-19 pandemic. As of February 28, 2022, a total of 474,001 cases and 4,140 deaths have been reported to the Puerto Rico Department of Health since March 2020^8^. The cumulative COVID-19 incidence rate is 14,813 cases per 100,000 population, and the COVID-19 mortality rate is 129.4 deaths per 100,000 population [8]. Residents of Puerto Rico are socially vulnerable to COVID-19 sequelae as the island has one of the highest prevalence rates for chronic diseases [9–14]. People with underlying chronic medical conditions have an increased risk of developing COVID-19-related symptom severity and mortality [11]. The rate of comorbid and multimorbid chronic conditions (e.g., cardiovascular diseases, obesity) is also higher in socially vulnerable communities [11, 12], and this tendency prevails in Puerto Rico. Therefore, the inclusion of community-based strategies among SVC is needed to reduce COVID-19-related health disparities.

The unique challenges posed by the COVID-19 pandemic require a team science approach coupled with community-based partnerships. The active role of the communities affected by the COVID-19 PHE is pivotal for the successful implementation of COVID-19 prevention strategies and the ultimate elimination of COVID-19-related health disparities. Providing community leaders with skills, abilities, and resources to understand and adapt to the changes caused by the COVID-19 pandemic with a research-driven approach is vital to community capacity building [15].

A theoretical consolidation of two existing well-known approaches, the Health Belief Model (HBM) [16] and Community Engagement (CE) principles [17], can provide a comprehensive framework to promote COVID-19 prevention practices among SVC in Puerto Rico. The HBM proposes that community leaders adopt health behaviors determined by two factors: (1) the perception of susceptibility to getting sick from COVID-19; and (2) the perception of disease severity. Trained community leaders can identify health disparities in their communities, which is one of the factors in the HBM model that is linked to disease severity. A strengthened CE approach allows community leaders to develop and implement promotion strategies, health education activities, and empower communities to direct efforts that can increase access to COVID-19 PCR tests and mitigate the impact of COVID-19 in SVC. By employing a CE approach, members of the affected communities will become the main actors of change by taking the leading role as lay health promoters with skills and resources that will contribute to enhance and promote COVID-19 preventive measures over time.

As a part of the COVID-19 response in PR, an Epidemiological Intelligence Community Network (EpI-Net) intervention was developed to increase COVID-19 testing and prevention practices among SVC on the island. EpI-Net is a community-based intervention based on CE principles of mutual benefits, trust, shared responsibility, respect for cultural values, collaboration, and capacity building. EpI-Net is an intervention with six components: (1) community leader capacity building; (2) syndromic surveillance for COVID-19; (3) molecular testing for severe acute respiratory syndrome coronavirus 2 (SARS-CoV-2) community campaign; (4) educational component; (5) linkage to case investigation systems; and (6) COVID-19 vaccine campaign. Our community leader capacity building component seeks to provide sustainable and long-lasting resources with an evidence-based approach to community leaders that will help them thrive during the COVID-19 pandemic and other public health emergencies. Local community leaders received public health trainings and collaborated with the research team during EpI-Net’s field interventions as part of the community leader capacity building component. This manuscript aims to evaluate EpI-Net’s community leaders capacity building component by assessing pre-post COVID-19 public health workshops’ tests’ score changes and satisfaction among trained community leaders.

## Methods

The EpI-Net team conducted a mixed-methods design for intervention trials [18], divided into two phases: (1) pre-implementation (qualitative component); and (2) implementation (EpI-Net intervention). The community leader’s capacity building component is the first part of phase 2. First, participants received in-person training related to public health topics and the COVID-19 pandemic by public health professionals and obtained quantitative data. After this step, community leaders also participated in a qualitative study with interview sessions (qualitative study not discussed in this article). Community leaders were also active participants in EpI-Net’s promotion strategies, information dissemination, education interventions, and COVID-19 testing interventions which will be evidenced by quantitative data. This manuscript is focused on a community leaders capacity building component to train community leaders from SVC. The overall hypothesis of the community capacity building component is that the integration of community leaders trained in COVID-19 prevention technology tools (EpI-Net) will result in high community leader retention levels over the intervention, increase community engagement strategies, and COVID-19 knowledge and prevention practices knowledge among the targeted SVC in Puerto Rico. Thus, the team assessed pre-post COVID-19 public health educational workshops’ test score changes among community leaders’ attendees.

Leveraging the community-based health promotion training infrastructure of Ponce Research Institute and the Research Centers for Minority Institutions Community Engagement Core (PHSU-RCMI) [19], the study team trained 24 lay community leaders from SVC in the Southern Region of Puerto Rico. The training program was focused on using COVID-19 epidemiological intelligence tools, data analyses, and implementation. The training consisted of four interactive group sessions in-person that covered the following topics adapted for community members: (1) principles of community engagement research; (2) fundamentals of public health emergencies; (3) COVID-19 prevention, promotion, and education; and (4) EpI-Net implementation training (See Table 1).

**Table 1:** Description of the Educational Workshops and Learning Objectives in the Training of EpI-Net Community Leaders

Educational Workshop	Learning Objectives
Workshop #1 Research and Community Participatory	1. Understand the basic concepts of research. 2. Define community participatory research. 3. Identify community engagement principles. 4. Establish community participatory strategies. 5. Develop a community engagement plan.
Workshop #2 Fundamentals in Public Health Emergencies	1. Know the basic concepts and fundamentals of public health emergencies. 2. Develop strategies in the principles of promotion, preparation, and response in public health emergencies. 3. Design a plan of activities for risk management, mitigation, and response in a public health emergency. 4. Identify tools and skills for individual and collective action for effective participation in public health risks and emergencies.
Workshop #3 Education, Promotion, and Prevention of COVID-19	1. Define COVID-19 disease. 2. Understand modes of transmission of COVID-19. 3. Recognize populations at risk for contracting COVID-19 diseases. 4. Understand the complications of COVID-19 disease. 5. Explain concepts of COVID-19 isolation vs. quarantine. 6. Know the testing for COVID-19 detection. 7. Understand the process of contact tracing. 8. Establish strategies for promotion, health education and prevention of COVID-19 disease in the community.
Workshop #4: Basic Concepts of Epidemiology and Epidemiological Intelligence Network (EpI-Net) Tools	1. Know the basic concepts of epidemiology for morbidity-mortality, distribution in time, place, and person. 2. Know epidemiological surveillance tools and the concept of epidemiological intelligence. 3. Recognize the importance of surveillance in public health. 4. Know strategies to realize COVID-19 detection testing in communities as support measures for disease control. 5. Apply the epidemiological tools in the EpI-Net Project.

Note: All four workshops were conducted in a two-day period. Definitions: COVID-19 = coronavirus 2019; EpI-Net = Epidemiology and Epidemiological Intelligence Network

### Community selection process

The team used an adapted version of the CDC SVI for Puerto Rico [6] and identified the eligible municipalities with the following criteria: (1) SVI vulnerability rates of 0.45 or above; and (2) located in the southern region of Puerto Rico. The following municipalities were included: Guánica (0.64), Guayanilla (0.62), Peñuelas (0.57), Santa Isabel (0.53), Ponce (0.51), and Juana Diaz (0.48). Once municipalities were identified, SVI was developed to identify those highly vulnerable communities (SVI of 0.45 or above).

### Eligibility criteria

A total of 21 SVCs were selected to participate in the EpI-Net intervention according to their SVI. Leveraging current community collaborations from research initiatives in our institution and through community site visits, the team identified possible candidates to participate and received training. The training sessions could only be attended by invitation, received in-person or by telephone. The community leaders’ recruitment goal was n = 5 community leaders for each selected municipality for an overall total of 30 community leaders trained by EpI-Net’s team. The inclusion criteria for the community leaders to be trained in EpI-Net activities were: 1) > 21 years of age; 2) living in a COVID-19 SVC according to the adapted SVI; 3) self-identified as a Latino/Hispanic; and 4) able to provide support for the EpI-Net implementation process in their communities.

### Data collection and analysis

For the study, selected community leaders completed an informed consent form. After the informed consent was obtained, their sociodemographic information was collected. The National Institutes of Health Common Data Elements provided by the Rapid Acceleration of Diagnostics-Underserved Populations (RADx-UP) Coordination and Data Collection Center (CDCC) were used to obtain this information. Pre- and post-tests were designed to observe changes in test scores for each session using evaluation questions developed by the public health expert offering the training (See Supplementary Materials). In addition, an overall evaluation of each workshop was used to assess the experience and satisfaction of the participants. Each workshop was evaluated on a scale from 1 to 4 using a 10-question survey at the end of each session.

Descriptive statistics, frequencies, and means were used for sociodemographic variables. A comparison between participants who completed the entire sessions of workshops and participants of some of the workshops was made using chi-square test. A two-sided Pearson chi-square was used for variables with an expected count equal or higher than five, and for variables with an expected count of less than five, a Fisher’s exact test was considered. The total possible score for each test was five. The percentages of correct answers were calculated for each topic workshop. Paired t-test was used to analyze pre-test scores to post-test scores. Paired t-test analysis was conducted to identify changes in pre-post-test scores and potential statistical differences (p < 0.05) with a two-sided p-value. Data were presented as mean and standard deviation. Statistical analysis was performed using R x64 4.1.0.

### Institutional review board (IRB)

IRB approval for RADx-UP Project #46 was obtained by the Ponce Research Institute (IRB Protocol #2011048329A004). Also, this project is part of the CDCC agreement for data sharing under federal regulations.

## Results

### Community leaders’ recruitment

The team invited 28 community leaders to participate in the training sessions. A total of 24 (85.7%) community leaders accepted the invitation to participate in the trainings. Twenty-four community leaders participated in at least one COVID-19 prevention technology tools training by EpI-Net, with 19 leaders (79.2%) completing the full training session, which consisted of four separate workshops.

### Sociodemographic characteristics

Among the 24 community leaders who participated in the training sessions, 70.8% were females, 58.3% reported having a private health insurance provider, and the mean age was approximately 54 years. Out of the 24 leaders, 17 (70.8%) reported never smoking or vaping, 2 (8.3%) leaders reported smoking some days, and 2 (8.3%) leaders reported smoking daily.

On March 12, 2021, the start date for data collection, 6 (25.0%) community leaders reported not being vaccinated for COVID-19; however, when interviewed in later months, community leaders (n = 20, 62.5%) reported being vaccinated for COVID-19. A total of 20 (83.3%) community leaders reported being tested for COVID-19 in the past. When comparing the community leaders who completed the full training series with those who did not, the average chronological age was 53.66 and 56.00, respectively. No statistically significant differences were identified at a 0.05 significance level for sociodemographic characteristics between the two comparison groups (See Table 2).

**Table 2:** Sociodemographic Characteristics and COVID-19-related Variables for Community Leaders Who Participated in EpI-Net’s Community-based Training

Variable	All participants		Completed full training^1^		Not completed full training^1^		p-value
	n	%	n	%	n	%	
Completed full training^1^							
Yes	19	79.2					
No	5	20.8					
Race							
Black or African American	4	16.7	3	12.5	1	4.2	-
White	10	41.7	9	37.5	1	4.2	0.516
Some other race	6	25.0	5	20.8	1	4.2	0.739
Prefer not to answer	1	4.2	1	4.2	0		0.566
NA	3	12.5	1	4.2	2	8.3	
Sex							
Male	4	16.7	4	16.7	0		-
Female	17	70.8	14	58.3	3	12.5	0.389
NA	3	12.5	1	4.2	2	8.3	
Health insurance							
Do not have health insurance	0						
Private insurance	14	58.3	13	54.2	1	4.2	-
Public insurance	7	29.2	5	20.8	2	8.3	0.204
NA	3	12.5	1	4.2	2	8.3	
Smoker status							
Never	17	70.8	15	62.5	2	8.3	-
Some days	2	8.3	2	8.3	0		0.663
Every day	2	8.3	1	4.2	1	4.2	0.166
NA	3	12.5	1	4.2	2	8.3	
COVID-19 vaccinated							
No	6	25.0	5	20.8	1	4.2	-
Yes	15	62.5	13	54.2	2	8.3	0.853
NA	3	12.5	1	4.2	2	8.3	
Tested for COVID-19							
No	1	4.2	1	4.2	0		-
Yes	20	83.3	17	70.8	3	12.5	0.694
NA	3	12.5	1	4.2	2	8.3	

Note: Significance level measured at 0.05. ^1^Completion of full training series is defined as a community leader who responded the pre- and post-test of all four workshops conducted by the EpI-Net team. COVID-19, coronavirus 2019; EpI-Net, Epidemiology and Epidemiological Intelligence Network

### Pre- and post-assessment results

Community leaders pre- and post-tests by workshop were compared by their mean total scores using a paired t-test. The mean score for the pre-test for workshop 1, “Foundations of Public Health Emergencies,” was 4.2 (SD: 0.9) out of 5.0, and for the post-test 4.6 (SD: 0.6) out of 5.0. The t-value when comparing the results was − 2.7, with a statistically significant two-sided p-value of 0.012. For workshop #2, “COVID-19 Education and Prevention”, the pre-test mean score (2.9 (SD: 0.7)) shows a statistically significant difference at a 0.05 level of significance (t-value = -8.6) from the post-test mean score (4.2 (SD: 0.7)). Workshop #3 pre and post-test, which focused on “Education, Promotion, and Prevention of COVID-19”, presented a statistically significant difference not likely due to chance (mean score for pre-test: 3.4; the mean score for post-test: 3.9; t-value: -2.6; p-value = 0.014). Workshop #4, “Foundations of Epidemiology Intelligence Tools,” was the only test that did not obtain a statistically significant difference (t-test: 0.4; p-value: 0.700) for the mean pre-test score (3.8 (SD: 1.3)) and the post-mean score (3.6 (SD: 1.4)) (See Table 3).

**Table 3:** Response Comparison for Pre- and Post-test for Community Leaders Participating in EpI-Net’s Community-based Training

Workshop	Pre-test		Post-test		Paired Sample t-Test	
	**Mean**	**Standard deviation**	**Mean**	**Standard deviation**	**t-value**	**p-value**
Workshop 1^1^	4.2	0.9	4.6	0.6	-2.7	0.012
Workshop 2^2^	2.9	0.7	4.2	0.7	-8.6	< 0.001
Workshop 3^3^	3.4	0.7	3.9	0.7	-2.6	0.014
Workshop 4^4^	3.8	1.3	3.6	1.4	0.4	0.700

Note: Significance level measured at 0.05 presented with a two-sided p-value. ^1^Workshop 1: Research and Community Participatory. ^2^Workshop 2: Fundamentals in Public Health Emergencies. ^3^Workshop 3: Education, Promotion, and Prevention of COVID-19. ^4^Workshop 4: Basic Concepts of Epidemiology and Epidemiological Intelligence Network (EpI-Net) Tools. EpI-Net, Epidemiology and Epidemiological Intelligence Network

### Participants’ workshops satisfaction results

Community leaders evaluated the workshops’ information, domain, interactions, and resources as excellent or good, with average participant scores for each question being 4 or higher than 3. All participants shared that they would recommend the sessions to future participants. All workshops received the highest possible score when asked what grade they would give each session. Workshop #2 obtained the lowest evaluation score (n = 3.9 out of 4, 99.13%), while workshop #1 obtained the highest evaluation score (n = 3.9 out of 4, 99.78%). Overall, scores reflected positive feedback from participants (See Table 4). Participants were asked for comments and suggestions for the workshops. Some participants shared, “[a]ll the topics were useful as they give us some tools to help the community,” “[t]he steps to carry out the investigation and the way to carry the message and contribute some improvement in the community”; “The topics are in accordance with the need that the communities have. The resources are very good”; “The Doctor presented the subject quite clearly. I became interested in the preparation of the “Family Plan,” now I consider it very important.” (See Supplementary Materials).

**Table 4:** Overall Community Leader’s Workshop Satisfaction Evaluations for the Information Received by the EpI-Net Team Presenters

Workshop	Total Leaders Who Completed the Survey	Overall Total Scores	Overall Average Score	Percentage Overall Average Score
Workshop 1^1,2^	23	91.8	3.9	99.8%
Workshop 2^1,3^	23	91.2	3.9	99.1%
Workshop 3^1,4^	22	87.8	3.9	99.8%
Workshop 4^1,5^	22	87.8	3.9	99.8%

Note: ^1^Each question was evaluated in a scale from 1 to 4, with Excellent = 4, Good = 3, Regular = 2, and Bad = 1. ^2^Workshop 1: Research and Community Participatory. ^3^Workshop 2: Fundamentals in Public Health Emergencies. ^4^Workshop 3: Education, Promotion, and Prevention of COVID-19. ^5^Workshop 4: Basic Concepts of Epidemiology and Epidemiological Intelligence Network (EpI-Net) Tools. COVID-19, coronavirus 2019; EpI-Net, Epidemiology and Epidemiological Intelligence Network

## Discussion

In general, EpI-Net’s community leaders capacity building training component was successfully carried out. An 85.7% of all invited community leaders participated in the training sessions. Overall, community leaders presented a change in pre-post-test educational scores in most training components, including community engagement research; public health emergencies; and COVID-19 prevention, promotion, and education. The assessment for pre-workshop #1 suggested that community leaders were familiarized with community-based research concepts.

However, our results still show increased post-changes scores for this workshop. The “Fundamentals of Public Health Emergencies” workshop showed the greatest post-test score changes, and participants shared the usefulness of the information for an island that is constantly under threat of natural disasters. For workshop #4, we did not find a significant pre-post score changes among participants. When evaluating training satisfaction, community leaders expressed positive feedback and helpfulness in the workshops they received. Despite not obtaining a statistically significance pre-post score changes for all four workshops in the training series, our results present the importance of including community leaders’ capacity building strategies by providing tools to help them face public health emergencies.

EpI-Net’s community leaders capacity building component pre-implementation taught us that there are at least five critical strategies for a successful implementation process: (1) integrate multiple community engagement levels into your approach; (2) provide tools and resources to SVC; (3) co-develop culturally relevant education materials for community members; (4) empower communities; and (5) integrate community leaders capacity building component as a tool to reduce health disparities and respond to PHE at the community level. These five strategies are explained in more detail below.

First, the current project reflects the experience of integrating multiple community engagement levels: (1) low level (e.g., community workshops); (2) intermediate level (e.g., outreach activities development); and (3) high level (e.g., academic-community partnership). Each level is a key component for the successful implementation process of this community-based intervention during a PHE [20].

Second, community-based interventions should provide tools and resources to SVC to face multiple structural barriers. Socioeconomic status, mistrust of research institutions, concerns about confidentiality, and lack of culturally-relevant information about research studies are known barriers to increasing participation of Hispanics in research activities [21–24]. Often, community members are not convinced of the benefits of research results for their communities. Also, research designs and recruitment plans may not be welcomed by community members if they reflect a lack of knowledge about cultural/social characteristics of the priority population. The EpI-Net intervention addressed these barriers by promoting the integration of community leaders in partnership with research activities through capacity building and community education components. The reciprocal communication channels between EpI-Net investigators and community members were an opportunity to enhance the CE principles of trust, shared responsibility, and collaboration.

Third, successful implementation depends upon co-developing culturally relevant education materials for community members. Each community has local diversity (e.g., age groups, educational level, chronic conditions, mental health conditions, etc.), and everyone belonging to these groups requires different dissemination strategies. Through EpI-Net, community leaders provide the team with multiple dissemination strategies to share COVID-19 prevention and educational materials according to community needs (e.g., flyer, poster, audio bus, community meetings, mobile unit visits, etc.). Furthermore, community leaders informed the EpI-Net team about the best ways to present COVID-19 data (e.g., statistics, community transmission levels, vaccination efficacy) in terms of relevancy for SVC.

Fourth, CE is a proven strategy for reducing health disparities [25–27]. Nevertheless, the success of CE is dependent upon empowering communities [28, 29]. Through EpI-Net, the community can build up and strengthen networks to address COVID-19-related challenges, as well as other health disparities at the community level. With the opportunity to see these contributions, an empowered community can better itself even further and be prepared for what may come.

Finally, providing opportunities for community capacity building is critical [30], and a key component to promote actions that can address health community needs to reduce health inequities. The COVID-19 PHE has resulted in dynamic responses and numerous community challenges, emphasizing the need to provide culturally relevant information and the critical importance of knowing how this information aligns with community needs. A community capacity building approach adapts resources to respond to SVC needs, thereby allowing SVC communities to develop their own strategies and create a plan to address the challenges related to the PHE. EpI-Net’s in-person training sessions allow the development of partnerships with community leaders. Maintaining communication with community leaders and integrating them into our research activities can translate into continuously sharing knowledge, tools, and resources for sustainability [31]. The next steps include measuring sustainability and if integrating community leaders to our interventions increases COVID-19 testing uptake and prevention practices.

Our intervention includes a set of limitations. First, not all community leaders of phase 1 completed the four workshop training sessions. Second, workshop #4 showed no statistically significant difference between the pre- and post-test. Therefore, a restructuring of the included information will be completed for future training sessions. Third, our sample only includes community leaders from socially vulnerable communities in Puerto Rico. Fourth, this intervention was conducted as part of a larger community-based research project. Future results, with a larger sample size, are expected to provide more inside into the impacts of community capacity building as a key component for public health interventions.

The community leader’s capacity building component and the training sessions offered by EpI-Net presented some challenges. We depend on the availability of community leaders. Some participants could not complete the entire training series due to other responsibilities. These community leaders will be invited to attend future sessions. Our results show the initial steps towards developing partnerships with local leaders and improving public health understanding with evidenced-based information developed and presented by public health experts.

## Conclusion and public health implications

Community leaders who participated in our intervention showed changes in pre-post scores of public health-related topics. However, they had difficulty understanding epidemiologic surveillance topics during a public health emergency, such as the COVID-19 pandemic. The training sessions created an environment of communication and engagement between community leaders and researchers, which allows addressing community needs and working to reduce health inequities. Investing in public health infrastructure is critical to providing a rapid response for future PHE. Such an investment should not only be made at the federal, state, and local level but the community level, as well. For the COVID-19 pandemic and future PHE responses, the integration of capacity building programs must be included in preparedness plans to assure cultural competence and relevance among communities that experience health disparities, such as SVC in Puerto Rico.

## Electronic supplementary material

Below is the link to the electronic supplementary material.

Supplementary Material 1

## Data Availability

The data that support the findings of this study are available from the EpI-Net Research Project, but restrictions apply to the availability of these data, and so are not publicly available. Data are however available from the authors upon reasonable request and with released permission from EpI-Net Research Project and the Ponce Research Institute, who can be contacted at epi-net@psm.edu.

## List of Abbreviations

CDC SVI: Centers for Disease Control and Prevention’s Social Vulnerability Index
CDCC: Coordination and Data Collection Center
CE: Community Engagement
COVID-19: coronavirus 2019
HBM: Health Belief Model
IRB: Institutional Review Board
PHE: Public Health Emergency
PR: Puerto Rico
RADx-UP: Rapid Acceleration of Diagnostics-Underserved Populations
SARS-CoV-2: severe acute respiratory syndrome coronavirus 2
SVC: Socially Vulnerable Communities
