# Supplementary material for: Epidemiological intelligence community network intervention: a community response for COVID-19 community transmission
Source: BMC Public Health. 2023 Jun 1;23:1044. doi: 10.1186/s12889-023-15727-3 (PMC10233188; doi:10.1186/s12889-023-15727-3)
Supplement: Supplementary file 1 — Supplementary Material 1 [file 12889_2023_15727_MOESM1_ESM.docx]

**Supplementary Materials: Epidemiological Intelligence Community Network Intervention: A Community Response for COVID-19 Community Transmission**

Melissa Marzan-Rodríguez^1,2,3^, Eida M. Castro-Figueroa^1,3^, Kamalich Muniz-Rodriguez^2^, Iris S. Martínez^1,2,3^, Luisa M. Morales^1,2,3^, Natasha Torres-Borrero^2^

^1^Public Health Program, Ponce Health Sciences University, Ponce, Puerto Rico.

^2^Ponce Research Institute, Ponce Health Sciences University, Ponce, Puerto Rico.

^3^School of Behavioral Sciences, Ponce Health Sciences University, Ponce, Puerto Rico.

**Contact information:** Melissa Marzan-Rodríguez; [mmarzan@psm.edu](mailto:mmarzan@psm.edu), Eida M. Castro-Figueroa; [ecastro@psm.edu](mailto:ecastro@psm.edu), Kamalich Muniz-Rodriguez; [kmuniz@psm.edu](mailto:kmuniz@psm.edu), Iris S. Martínez; [irismartinez@psm.edu](mailto:irismartinez@psm.edu), Luisa M. Morales; [lmorales@psm.edu](mailto:lmorales@psm.edu), Natasha Torres-Borrero; [ntorres@psm.edu](mailto:ntorres@psm.edu).

**Pre- and post-test for EpI-Net’s community leaders capacity building component**

Workshop #1: “Research and Community Participatory”

1. Research is a scientific approach to answer questions from communities.
   1. True
   2. False
2. The steps for the investigation are:
   1. Data Collection, Analyze, Interpret, Conclude
   2. Identify problem, Analyze, Interpret, Conclude
   3. Identify problem, Data Collection, Analyze, Interpret, Conclude
   4. Observe, Interpret, Conclude
3. An example of observing a problem in the community is:
   1. People at higher risk of complications from COVID-19 live on 23 street
   2. No COVID-19 testing campaigns have been carried out
   3. Little use of masks in young people aged 13-16
   4. All of the above
4. Which of the following answers is considered a pillar in community participation?
   1. Location
   2. Leadership and organization
   3. Individualism
   4. None of the above
5. Which of the following is NOT a value of the community link?
   1. Opening
   2. Respect for Diversity
   3. Profit
   4. Training and empowerment

Workshop #2: “Fundamentals in Public Health Emergencies”

1. The ability of communities to prepare for, resist, and recover from both short- and long-term public health incidents is known as:
   1. Community readiness
   2. Community response
   3. Community emergency
   4. Community recovery
2. In case of interruption of the water service, it is recommended to boil the water for a minimum of _____ before consuming it, to avoid the transmission of diseases.
   1. 1 minute
   2. 3 minutes
   3. 5 minutes
   4. 10 minutes
3. The emergency management phase of ___________________ consists of long-term activities and programs, beyond the initial crisis period of an emergency or disaster.
   1. Preparation
   2. Response
   3. Recovery
   4. Mitigation
4. The first response to an emergency is always given at the _____________ level
   1. International
   2. National
   3. State
   4. Local
5. A landslide is considered a type of disaster:
   1. Natural
   2. Technological
   3. Accidental
   4. Terrorist

Workshop #3: “Education, Promotion, and Prevention of COVID-19”

1. The mode of transmission of COVID-19 is through droplets of saliva that contain the virus when people cough, sneeze, or talk.
   1. True
   2. False
2. Which of the following does NOT belong to the group of people with the highest risk of becoming seriously ill with COVID-19:
   1. Young people
   2. People with asthma
   3. People with down syndrome
   4. People with obesity
3. Isolation keeps a person who has been in close contact with someone who has COVID-19 away from others:
4. True
5. False
6. Health promotion strategies are:
   1. Communication, individual participation, empowerment
   2. Communication, participation of institutions, literacy for health
   3. Communication, education, citizen participation, consultation (agreements)
   4. Communication, schools, universities
7. Generates tools for individuals and populations to understand, transform, and use knowledge and resources at the service of satisfying needs, developing potential, solving problems and conflicts.
   1. Communication
   2. Education
   3. Citizen participation
   4. Concertation (agreements)

Workshop #4: “Basic Concepts of Epidemiology and Epidemiological Intelligence Network (EpI-Net) Tools”

1. The epidemic curve is a tool to identify:
   1. Weather
   2. Place
   3. Person
   4. None of the above
2. Geographic location is a variable associated with:
   1. Weather
   2. Place
   3. Person
   4. None of the above
3. The disease is the result of the interaction of:
   1. Time, place and person
   2. Host, agent and environment
   3. Community vectors
   4. None of the above
4. The systematic and continuous observation of the frequency, distribution and determinants of health events and their trends in the population refers to:
   1. Epidemiology
   2. Epidemiological surveillance
   3. Epidemiological intelligence
   4. Epidemiological incidence
5. Which of the following is NOT a basic criterion for carrying out epidemiological surveillance?
   1. A public health problem
   2. Ease of acquiring data
   3. There are public health interventions (treatments, prevention)
   4. The entire population must be vulnerable

**Community leaders’ evaluation of EpI-Net’s workshops**

The evaluations community leaders completed at the end of each workshop provided a space to write comments or suggestions for the material shared in each presentation, and to share which topic or activity they found more useful. Not all participants provided written comments, but those who did, wrote them in Spanish. Table SM-Table 1 presents a literal translation of the comments left by participants in no particular order.

**SM-Table 1.** Responses to opened ended questions in the evaluation forms for each workshop in EpI-Net’s community leaders training sessions

| Workshop number | **Question 1: What topics or activities in the session did you like the most or find most useful?** | **Question 2: Any additional comments and/or suggestions?** |
| --- | --- | --- |
| Workshop 1 | All the topics were useful as they give us some tools to help the community. | Everything flows very well |
|  | All excellent | Excellent in emphasizing the importance of the "backpack" |
|  | The toolbox | The themes are in accordance with the need that the communities have. The resources are very good. |
|  | Community Engagement and Strategies | Very good information and dynamic I congratulate you |
|  | The examples used and the theme made it clear and concise | Excellent thanks =) |
|  | All very interesting, clear for community leaders | If they can contact other leaders |
|  | Toolbox explanation exercise | Excellent resource |
|  | Listen for problems and possible investigation | Thank you! |
|  | All | Excellent presentation |
|  | The steps to carry out the investigation and the way to carry the message and contribute some improvement in the community. | Very well! Super!!! |
|  | All very excellent | Excellent resource |
|  | Community engagement strategies | Bring educational workshops to marginalized and vulnerable communities |
|  | Research and Community Engagement | Bring more workshops and education to communities |
|  | Research | Excellent resource :) |
|  | Communal health | None |
|  | They were all very well explained-excellent | I suggest an emergency management workshop for community leaders |
|  | All |  |
|  | Other research programs available on Epinet. |  |
|  | The dynamic that was established |  |
|  | The simplicity of the resources |  |
|  | They are all useful topics |  |
| Workshop 2 | The explanation in preparation in case of emergency. | Resources are well targeted |
|  | The topics were clearly presented and help us to be able to educate the community. | Everything's fine |
|  | The process of working an emergency | This type of training is very necessary and efficient to expand and have this knowledge that can save lives. Thank you!! |
|  | Phases/Very interesting backpack | I like to go to workshops that are to the point and are not monotonous. |
|  | The preparation and recovery | Very good the issues of the backpack and preparation |
|  | All | Very well presented. Simple, short and direct |
|  | Backpack | May this orientation reach more people. |
|  | The Doctor presented the subject quite clearly. I became interested in the preparation of the "Family Plan", now I consider it very important. | Excellent resource |
|  | Create an emergency plan at home | Give it to all the leaders of the municipality |
|  | All very good and interesting information | That can be shared with school communities. |
|  | All! | Excellent presentation |
|  | Emergency backpack, hurricane-earthquake prevention measures | Excellent, very good workshop |
|  | Research and Community Engagement | Excellent work |
|  | Emergency plan | Educate the population |
|  | Disaster and emergency prevention and management | Excellent resource and very pertinent to the topic for our communities |
|  | The importance of being prepared for an emergency |  |
|  | All |  |
|  | The interaction with the resource was excellent |  |
| Workshop 3 | Creating epidemiological networks | Good information and team |
|  | All | Very good everything, I learned a lot. Thank you |
|  | Open questions | Excellent resource, very dynamic, sincere and with a lot of knowledge |
|  | Epidemic surveillance | It was the way i like it, short, simple and concise |
|  | All the themes | Better time management |
|  | All | Excellent |
|  | Everything detailed and simple | Thank you! |
|  | Health promoter | Excellent |
|  | All | Excellent |
|  | Community health promotion | Excellent team |
|  | All | Everything is very interesting and I have learned a lot. Thanks. |
|  | All | Very interesting, appropriate topics |
|  | Prevention | If they do it better they damage it, excellent team |
|  | Information about contagion covid-19 | They are definitely amazing |
|  | All | Being facilitators in the com. And center frequently. I congratulate you for your work, Dlb. |
|  | Group participation | Excellent both themes |
|  | Strategies and everything related to covid |  |
|  | I liked it all |  |
|  | I liked them all |  |
|  | All |  |
|  | Classic epidemiological variables |  |
|  | Explanation, tests and vaccination |  |
| Workshop 4 | Epidemic curve | Excellent resource |
|  | All | The place is very appropriate to carry out the workshop. I had already taken the workshop |
|  | The epidemiological curve | Thank you! |
|  | All | Excellent presentation of a lot of knowledge for me |
|  | Triad, Epidemiology, Basic criteria to establish an epidemiological health surveillance system | Interesting |
|  | All | Excellent |
|  | Being part of the Epi-NET team | Excellent Take these workshops to the communities |
|  | Epidemiological triad |  |
